# Multisystem Benefits of GLP-1 Microdosing: A Narrative Review

**DOI:** 10.7759/cureus.113635

**Published:** 2026-07-30

**Authors:** Mia A Panlilio, Natalie Piserchio, Mercedes Hennessey, Kayla Torres, Sana Khan, Lucas Choy

**Affiliations:** 1 College of Osteopathic Medicine, Rocky Vista University, Englewood, USA; 2 Internal Medicine, Sky Ridge Medical Center, Lone Tree, USA

**Keywords:** glp-1, glp-1 agonists, metabolism, microdosing, obesity, semaglutide, tirzepatide

## Abstract

Glucagon-like peptide-1 (GLP-1) is an endocrine hormone that plays a role in lowering blood glucose levels and promoting weight loss. GLP-1 receptor agonists (GLP-1 RAs) are synthetic peptides that mimic the activity of GLP-1, and the recent development of GLP-1 RA medications has been a major historical advancement in the treatment of diabetes and obesity. Despite the rapid development of GLP-1 RA medications, there is a very high demand for the limited supply of these drugs, resulting in high medication costs and significant care gaps among prescribed patients. This shortage has forced providers to be more strategic and creative with their treatments in order to support therapeutic continuity, which has led to an increased utilization of medication microdosing. This article aims to summarize available evidence on the effects of GLP-1 RAs and the possible benefits of long-term microdosing GLP-1 RA medications.

## Introduction and background

Obesity is a growing public health issue, both in the United States (US) and worldwide, due to a multitude of interacting factors such as an individual’s genetics, neurobiology, and eating behaviors [1]. It is defined as an individual whose body mass index (BMI) is greater than or equal to the 95th percentile based on the sex-specific BMI-for-age 2000 CDC Growth Charts [2]. In the US, the prevalence of generalized obesity (BMI > 30 kg/m^2^), extreme obesity (BMI > 40 kg/m^2^), and central obesity continues to rise in children and adults [3]. 

The most recent National Health and Nutrition Examination Survey (NHANES) data from August 2021 to August 2023 showed an estimated 21.1% of US children and adolescents ages two to 19 who have generalized obesity, including 7.0% with severe obesity, and another 15.1% were overweight [4]. Data also showed the prevalence of generalized obesity among US adults to be 40.3%, with no significant differences between men and women; the prevalence of severe obesity to be 9.4%, notably being significantly higher in women than men; and the prevalence for overweight adults to be 31.7% [5]. The associated health risks of obesity increase in a dose-dependent manner across BMI classes, with the most pronounced risks in cardiovascular disease, type 2 diabetes, and cancer [6].

Current treatments for obesity and associated conditions

Most obesity treatment includes interventions, such as behavioral interventions, nutrition counseling, physical activity, pharmacotherapy, and metabolic/bariatric procedures, with more comprehensive obesity treatment plans combining multiple interventions for individual patient needs [6].

Behavioral interventions, such as calorie restriction, physical activity, and individual and group counseling, were studied in the Look AHEAD (Action for Health in Diabetes) trial, which selected for adults with obesity and type 2 diabetes. Their results include a mean one-year weight loss of 8.6% and a mean 4.7% maintained weight loss at eight years, with approximately 27% of individuals maintaining ≥10% weight loss at eight years. The key to maintained weight loss in this setting was adherence, with 89% of alive participants completing the study [7]. A systematic review of adherence rates of behavioral intervention for weight management found dropout rates of up to 62% across several studies [8]. 

Older pharmacological interventions include phentermine, topiramate, naltrexone-bupropion, orlistat, or combination formularies, such as phentermine-topiramate. These older options yield a one-year weight loss of up to 10% and some as low as 3% [9]. These medications also come with adverse effect profiles, leaving patients with undesirable symptoms. For example, phentermine is known to commonly cause insomnia, anxiety, irritability, and palpitations [10]. Topiramate is known to commonly cause paresthesias, hypoesthesias, and psychomotor disturbances [11]. 

Despite these multiple avenues for individualized treatment, many patients still struggle to successfully manage their obesity with optimized weight loss but without deleterious adverse effects. This has pushed the scientific community to identify more effective solutions.

GLP-1 receptor agonists 

Glucagon-like peptide-1 (GLP-1) is a naturally produced endocrine hormone that plays a role in lowering blood glucose levels and promoting weight loss [1]. GLP-1 receptor agonists (GLP-1 RAs) are synthetic peptides that mimic the activity of GLP-1, and the fairly recent development of GLP-1 RA drugs has been a major advancement in the treatment of diabetes and obesity. 

In 2005, the first FDA-approved GLP-1 RA, exenatide, was released to treat type 2 diabetes and was available in a twice-daily formulation. Shortly after exenatide’s development, the drug class expanded to include once-daily formulations (liraglutide and lixisenatide) and eventually the current once-weekly formulations (dulaglutide, exenatide extended-release, and semaglutide) [12]. More recently, oral semaglutide formulations have been made available, and they are the first oral GLP-1 RA formulations. 

The shift beyond GLP-1 RA's primary indication of diabetes treatment to include weight loss occurred in 2015, when liraglutide was approved for use in chronic weight management [1]. At the moment, liraglutide, semaglutide, and tirzepatide are approved for use for chronic weight management in adults, and many other GLP-1 RA formulations are under development for potential use in many other indications that extend beyond diabetes and weight reduction [13].

Despite the rapid development of GLP-1 RA medications, there currently is a very high demand compared to the limited supply of these drugs, which has resulted in high costs of the medication and significant care gaps among prescribed patients. This has forced providers to be more strategic with their treatments in order to support therapeutic continuity, leading to an increased utilization of medication microdosing. 

Definitions and practical forms of microdosing

Microdosing first appeared in the late 1990s as a method of assessing pharmacokinetics in humans before a drug’s full phase I clinical trials were conducted [14]. When using microdosing in these pharmacokinetic studies, a sub-pharmacologically active dose of a given drug is administered, and human plasma samples are collected and analyzed for parent drug or metabolites. 

Since the development of the laboratory microdosing method, it has also been utilized clinically as a treatment strategy where a patient takes a medication in smaller, fractional doses [15]. By dosing in these smaller, fractional amounts of a given medication, patients can still receive some therapeutic benefits while concurrently extending the use of their medication, increasing drug tolerability, and minimizing side effects. Microdosing additionally further tailors treatment for each patient by allowing for dose reductions without needing a refill or new prescription. GLP-1 RAs are currently available in either a multi-dose pen or multi-dose vial, which makes microdosing an accessible treatment option for patients. 

This article aims to summarize available evidence on the effects of GLP-1 RAs, as well as the documented effects of microdosing the medications, and discuss the possible benefits of microdosing GLP-1 RAs long-term.

Methods

A non-systematic literature search was performed across electronic databases, including PubMed/MEDLINE, Scopus, and Google Scholar. Searches for articles were conducted using a combination of keywords and phrases related to GLP-1 RAs and their various effects across different body systems, including “GLP-1 microdosing,” “GLP-1 microdosing and metabolism,” and “GLP-1 and systemic effects.” Only articles that were from peer-reviewed journals and available in an English translation were included. There were no restrictions on the publication date or article type, and no formal reporting guidelines were followed. Search terms were refined throughout the review process to capture additional relevant studies, and reference lists of selected articles were manually screened to identify further articles of interest. Articles were selected based on their relevance to the topic and their contribution to the understanding of GLP-1 RAs and dermatologic conditions and if they were available in the English language. If the studies did not meet these criteria, they were excluded from this narrative review.

## Review

Standard GLP-1 RA mechanisms and established clinical benefits

GLP-1 RAs agonize the GLP-1 receptor to promote satiety and modulate energy intake. In addition to their effects on appetite, GLP-1 RAs improve glycemic control through glucose-dependent insulin secretion and suppression of glucagon, which contributes to improved metabolic regulation in patients with type 2 diabetes mellitus (T2DM) [16].

From a 2026 umbrella review of clinical outcomes across multiple diseases done by Kong [17], GLP-1 RAs showed trends toward improvements in endocrine and metabolic, cardiovascular, renal, and respiratory outcomes, cognitive function, with a potential reduction in fracture risk and all-cause mortality in certain populations, such as those with T2DM or polycystic ovarian syndrome (PCOS). These findings suggest that the effects of GLP-1 RAs can extend beyond weight loss and benefit multiple organ systems. 

The primary therapeutic efficacy of GLP-1 RAs is rooted in their ability to mimic endogenous incretin hormones, which modulate energy homeostasis through central and peripheral pathways. Early physiological studies established that GLP-1 infusion significantly centrally promotes satiety by acting on the hypothalamus and peripherally suppresses energy intake in humans, in part through delayed gastric emptying [18]. In this study, GLP-1 infusion was associated with reduced short-term caloric intake during meals when compared to a saline placebo. Importantly, there were no differences in the subjective ratings of taste, visual appeal, smell, aftertaste, or palatability of the meal [18]. Modern long-acting formulations have refined this mechanism, providing sustained receptor activation that leads to clinically significant weight reduction and improved glycemic control. 

Beyond weight loss, the clinical utility of GLP-1 RAs has expanded to encompass a broad range of systemic improvements. A comprehensive 2026 umbrella review by Kong [17] highlighted favorable trends across endocrine, metabolic, cardiovascular, renal, and respiratory outcomes. Notably, this evaluation suggested potential secondary benefits, including enhanced cognitive function, a reduction in fracture risk, and a decrease in all-cause mortality among specific patient populations. 

Evidence from a systematic review and meta-analysis of 21 trials including nearly 100,000 patients demonstrated that GLP-1 RAs significantly reduced major adverse cardiovascular events (MACEs), cardiovascular mortality, and all-cause mortality compared with controls [19]. Specifically, this analysis found reductions in serious adverse events, such as myocardial infarction (-15%) and heart failure hospitalization (-15%), with the use of GLP-1 RAs. Gastrointestinal (+63%) and gallbladder-related (+26%) adverse effects were increased. These effects were mostly consistent across subgroups including patients with and without diabetes, obesity, chronic kidney disease, and heart failure [19]. 

Furthermore, a systematic review and meta-analysis of 10 randomized controlled trials including 71,351 patients with type 2 diabetes found that long-acting GLP-1 RAs reduced MACEs by 14% (HR 0.86; 95% CI 0.81-0.90), hospitalization for heart failure by 14% (HR 0.86; 95% CI 0.79-0.93), and all-cause mortality by 12% (HR 0.88; 95% CI 0.82-0.93) [20]. In addition, GLP-1 RAs improved the composite kidney outcome by 17% (HR 0.83; 95% CI 0.75-0.92), including progression to kidney failure or sustained decline in eGFR. These benefits were consistent across both subcutaneous and oral formulations, with no increased risks of severe hypoglycemia, retinopathy, or pancreatic events [20]. 

An umbrella review by Yeo [16] provided a comprehensive evidence map across 432 randomized controlled trials and 65 health outcomes, demonstrating that GLP-1 RAs are associated with improvements in glycemic control, body weight, and several cardiovascular and renal outcomes. Notably, reductions in HbA1c (0.83 [0.71-0.97]) and body weight (eOR, 0.46 [95% CI, 0.36-0.60]) were supported by moderate- to high-certainty evidence. Cardiovascular outcomes, such as risk of heart failure (eOR, 0.71 (95% CI, 0.64 to 0.79)) and peripheral artery disease (eOR, 0.75 (95% CI, 0.67 to 0.84)) were reduced with low-certainty evidence. In addition, evidence regarding renal benefits (eOR, 0.76 (95% CI, 0.66-0.87)) had low certainty. This review also consistently identified increased gastrointestinal adverse effects, including nausea (9.62 (4.60-20.10)), dyspepsia (4.85 (1.52-15.45)), and constipation (3.39 (1.54-7.47)) as limitations of therapy with moderate-to-high certainty [16].

In a double-blind, phase 2 trial studying the efficacy and safety of semaglutide in patients with nonalcoholic steatohepatitis (NASH), 320 patients (of whom 230 were diagnosed stage F2 or F3 fibrosis) were randomly assigned to receive semaglutide at dosages of 0.1 mg (80 patients), 0.2 mg (78 patients), or 0.4 mg (82 patients) or to receive a placebo (80 patients) [21]. NASH resolution was achieved with no worsening of fibrosis at percentages of 40% in the 0.1 mg group, 36% in the 0.2 mg group, 59% in the 0.4 mg group, and 17% in the placebo group (P < 0.001 for semaglutide 0.4 mg vs. placebo). Fibrosis stage improvement was noted in 43% of the patients in the 0.4 mg group and in 33% of the patients in the placebo group (P = 0.48). A primary driver of NASH resolution independent of direct receptor agonism is the reduction in visceral adiposity, which directly decreases the portal free fatty acid flux to the liver. Hepatic tissue also does not express the GLP-1 receptor, so the study proposed the improvements in NASH were likely due to positive effects on weight and insulin resistance, and reductions in metabolic dysfunction, lipotoxic effects, and inflammation that resulted in downstream benefits for the liver [21,22].

Relating to the potential anti-inflammatory benefits of GLP-1 RAs, a case report of a 34-year-old woman diagnosed with type 2, stage III lipedema, characterized by uneven skin with mattress-like indentations from the hips to knees and larger, walnut-sized nodular fat deposits in the subcutaneous tissue, demonstrated positive improvements in her condition after 30 days of low-dose (1.5 mg weekly) tirzepatide [23,24]. As seen in its cardiovascular benefits, tirzepatide has systemic anti-inflammatory effects in response to low-grade inflammation. Some evidence shows that this anti-inflammatory effect is due to reductions in the pro-inflammatory markers hsCRP (high-sensitivity C-reactive protein) (mean difference (MD): -32.9; 95% confidence interval (CI): -33.6 to - 32.2; I² = 15.3%) and IL-6 (MD: -17.8; 95% CI: -24.3 to - 11.3; I² = 1.6%), which likely played a role in reducing the inflammatory burden seen in this patient’s case of lipedema [25]. Additional studies are needed to validate this effect, but this case report supports the overall anti-inflammatory benefits of tirzepatide treatment.

Regarding neurological conditions, previous research has shown that patients with T2DM have an increased risk of developing Parkinson’s disease (PD) [26]. Although biological mechanisms have not yet been fully defined, evidence suggests that insulin resistance can contribute to the increased production of reactive oxygen species, which can then cause oxidative stress, damaging the dopaminergic neurons in the substantia nigra. Other studies have shown that diabetic patients being treated with GLP-1 RAs have a reduced risk of developing PD, which suggests potential neuroprotective benefits [27,28].

A systematic review by Helal et al. [29] summarized the literature relating to the impacts of GLP-1 RAs in PD management, using the motor experiences of daily living (as assessed by the Movement Disorder Society-Unified Parkinson's Disease Rating Scale (MDS-UPDRS) Part II), motor impairment in PD (as assessed by the MDS-UPDRS Part III), motor complications (as assessed by the MDS-UPDRS Part IV), and the incidence of gastrointestinal and systemic side effects as primary outcomes for analysis. The MDS-UPDRS is a clinical tool with 65 questions divided into four parts that are used to measure, score, and track the severity and progression of Parkinson's disease [30]. Part I is related to the non-motor experiences of daily living, Part II is on the motor experiences of daily living, Part II is motor examination, and Part IV is related to motor complications. The article by Helal et al. [29] found that GLP-1 RAs significantly improved motor function, as evidenced by the score for the MDS-UPDRS Part III in the ON state (mean difference = − 2.88; p = 0.01; I2 = 30%), with no significant different for MDS-UPDRS Part II (mean difference = − 1.85; 95% CI − 3.72 to 0.02; p = 0.05) and Part IV (mean difference = − 0.30; 95% CI − 1.02 to 0.41; p = 0.41). Despite this improvement in motor function, GLP-1 RAs also were associated with a higher incidence of adverse events across all safety outcomes, with significant differences in dyspepsia, vomiting, and constipation, and non-significant differences in diarrhea, headache, and fatigue.

Tolerability, access, and rationale for individualized dosing

Understanding the diverse systemic benefits of GLP-1 RAs is critical when considering a microdosing approach. If these medications provide multi-organ protection even at submaximal doses, microdosing may hypothetically offer a viable pathway for patients to maintain metabolic stability and cardiovascular risk reduction while avoiding the dose-dependent gastrointestinal distress that often leads to treatment discontinuation and saving in medication costs.

Evidence from large meta-analyses and umbrella reviews demonstrates that GLP-1 RAs exert clinically meaningful effects across multiple domains, including reductions in HbA1c, body weight, cardiovascular events, and kidney-related outcomes [16,19,20]. Gastrointestinal side effects remain a primary limitation of long-term GLP-1 RA therapy and are one of the leading causes of discontinuation. 

A narrative review by Yılmaz and Bastemir [31] examining the reported side effects of GLP-1 RAs found that GI adverse effects remain the most common limitations to long-term therapy. Similar to the findings of previously mentioned studies, the most commonly reported GI adverse effects were nausea, vomiting, diarrhea, and constipation. More serious adverse effects such as delayed gastric emptying, gastroparesis-like symptoms, intestinal obstruction, biliary disease, and pancreatic safety concerns were variably reported as well; however, there is inconsistent evidence regarding these serious complications.

As suggested in a study by Petri et al. [32], tolerability often improves over time, indicating that lower or fractional dosing may support continued therapy and improve long-term adherence. Microdosing introduces a tolerability-first framework, allowing clinicians to tailor dosing to individual patient sensitivity and reduce early treatment barriers. This approach may be valuable for patients who are highly sensitive to adverse effects or demonstrate an exaggerated pharmacological response to a standard drug dose, i.e., “hyper responders.” Mechanisms involved in this exaggerated pharmacological response may be due to altered drug metabolism, altered receptor sensitivity or downstream signaling, or disease-related response variability [33]. 

Current recommended dosing regimens

For GLP-1 RAs like semaglutide and liraglutide, gradual dose titration is recommended to minimize risk of adverse gastrointestinal effects. Based on recommendations from the 2022 American Gastroenterological Association (AGA) Clinical Practice Guideline on Pharmacological Interventions for Adults With Obesity, semaglutide dosing for obesity starts at 0.25 mg weekly for the first four weeks, followed by doses of 0.5 mg, 1.0 mg, and 1.7 mg weekly every four weeks at each dose, until the maintenance dose of 2.4 mg is reached after 16 weeks [9]. As an antidiabetic therapy, semaglutide can be dosed up to 2.0 mg as a weekly subcutaneous injection. Liraglutide follows a shorter titration regimen for obesity, with the AGA recommending starting with 0.6 mg daily for the first 7 days, followed by doses of 1.2 mg, 1.8 mg, and 2.4 mg daily every seven days at each dose until the maintenance dose of 3.0 mg is reached after four weeks [9]. As an antidiabetic therapy, liraglutide can be dosed up to 1.8 mg as a daily subcutaneous injection. 

For tirzepatide, dosing is identical for both obesity and type 2 diabetes management [34]. The recommended starting dose is 2.5 mg once weekly for both indications, and it should then be increased to 5 mg once weekly after four weeks. Dosage should be increased in 2.5 mg increments every four weeks until a patient’s individualized maintenance dose is reached, with 15 mg being the maximum recommended weekly dose.

Despite these general recommended dosing strategies, provider clinical judgment is recommended for adjusting the titration schedule as needed based on each patient’s response so therapy can be tailored appropriately.

Comparison of incretin-based therapies

Semaglutide is a selective GLP-1 RA, whereas tirzepatide is a dual glucose-dependent insulinotropic polypeptide (GIP) and GLP-1 receptor agonist [35]. Both agents are potent incretin mimetics; however, their pharmacological profiles differ in ways that may influence microdosing strategies. 

GIP is the primary incretin in healthy individuals that potentiates glucose-dependent insulin secretion [35]. In T2DM, the insulinotropic effect of GIP is blunted, but pharmacological GIPR agonism may partially restore this response. GIP receptors are also highly expressed in the central nervous system hypothalamic feeding centers and white and brown adipose tissue, and agonism of these receptors has been shown to suppress appetite and food intake via GABAergic neurons, as well as enhance the lipid-buffering capacity of adipocytes, which promotes glucose and lipid uptake/disposal in subcutaneous fat rather than ectopic depots (liver and muscle). Similar to GIP, glucagon suppresses appetite and additionally increases energy expenditure through hepatic glucagon receptor signaling via persistent cAMP/PKA activation [35]. Glucagon signaling also suppresses de novo lipogenesis, enhances mitochondrial fatty acid β-oxidation, and increases follistatin secretion, collectively reducing hepatic steatosis. Lastly, amylin can also contribute to central satiety by acting on the area postrema and nucleus of the solitary tract (hindbrain) to induce satiety and satiation [35]. It can also delay gastric emptying and enhance glucagon suppression. Furthermore, Amylin has synergistic effects with leptin by improving leptin responsiveness, something that is typically impaired in obesity.

As GLP-1 RA drugs have evolved, the drug class now includes newer, next-generation formulations that target multiple receptors simultaneously. These advancements show greater metabolic efficacy, particularly in weight reduction and glycemic control compared to traditional GLP-1 RA monotherapy [36]. Other entero-pancreatic hormone combinations that act as dual (GIP/GLP-1) or triple (GIP/GLP-1/glucagon) receptor agonists have demonstrated greater reductions in weight and improvements in glycemic control compared to GLP-1 RAs alone. Specific multi-receptor combinations include cagrisema (GLP-1/amylin RA) and retatrutide (GIP/GLP-1/glucagon RA), which are newer drugs that have entered phase 3 trials for the treatment of obesity [37].

Early data on these multi-receptor agents suggest they could lead to even greater reductions in weight compared to tirzepatide. These enhanced effects are attributed to complementary mechanisms across metabolic pathways, including increased energy expenditure, suppression of energy intake, and improved lipid metabolism [36]. However, these multi-receptor agents are also associated with a higher incidence of gastrointestinal adverse effects, and ongoing research aims to optimize dose-escalation regimens to improve tolerability. 

Recent data from the SURMOUNT-5 trial demonstrated that tirzepatide may lead to superior weight reduction compared to semaglutide at maximum tolerated doses [38]. The dual-agonist mechanism of tirzepatide also introduces a more complex pharmacokinetic landscape. In real-world assessments, tirzepatide has shown significant effectiveness in individuals without type 2 diabetes, yet the necessity for precise titration remains paramount to mitigate adverse events [39]. From a microdosing perspective, the multi-dose vial or pen delivery systems for GLP-1 medications allow for clinician-guided fractional dosing. While semaglutide has a well-documented exposure-response relationship where lower doses (0.5 mg) still provide significant glycemic and metabolic benefits [32], tirzepatide’s dose-dependent efficacy appears more linear, suggesting that microdosing may be particularly useful for patients who are "hyperresponders" or those sensitive to the GIP-mediated effects.

Direct evidence for microdosing or low-dose maintenance

Studies specifically investigating the effects of microdosing GLP-1 RAs are extremely limited at this point in time; however, the AGA notes that based on evidence from dose-escalation trials, some patients achieve a strong therapeutic response at submaximal doses and can continue that dose on a long-term basis [34]. A pooled analysis of four phase III trials investigating once-weekly semaglutide doses 0.5 and 1.0 mg revealed that drug tolerability improved over time and sensitivity to gastrointestinal adverse events decreased with prolonged exposure [32]. More recently, a prospective multicenter observational study on the effectiveness of low-dose tirzepatide in nondiabetic adults with obesity showed notable improvements in mean body weight, BMI reduction, as well as beneficial decreases in HbA1c, LDL cholesterol, and triglyceride levels [39]. 

Safety and regulatory considerations

Current devices for drug administration typically only offer preset injectable amounts, or suggest that the injectable be used within a certain timeframe after opening. Taking the possibility of microdosing into consideration, newer devices could potentially be created specifically for microdosing utilization, thus decreasing the risk of sterility and safety concerns. 

Evidence gaps and future research

The increasing use of microdosing reflects a broader shift toward precision medicine in metabolic care. While current titration guidelines provide a standardized framework, they do not account for interindividual variability in response to incretin-based therapies. Future research should aim to formally evaluate microdosing strategies through prospective and longitudinal studies, with particular attention to long-term metabolic, cardiovascular, and adherence outcomes. Until such data are available, individualized clinical judgment remains essential in balancing efficacy, tolerability, and access to optimize patient care.

## Conclusions

The clinical landscape of GLP-1 RA therapy is shifting from a rigid, dose-escalation model toward a more flexible, individualized approach. Evidence presented in this review suggests that therapeutic benefit from GLP-1 RAs is not limited to a single outcome or body system. This raises the possibility that maintaining some degree of GLP-1 receptor activation may preserve clinically meaningful metabolic and cardiovascular effects in some patients. Rather than prioritizing rapid escalation to a maximum tolerated dose, a more gradual, individualized approach may allow patients to maintain therapeutic exposure while minimizing treatment burden and adverse effects. 

Although microdosing originated as a pharmacokinetic tool for early-phase drug development, it has emerged as a pragmatic strategy in modern clinical practice. This shift is driven not only by the need to navigate drug shortages and rising costs, but also by an evolving understanding of the multi-system benefits of GLP-1 signaling across metabolic, cardiovascular, and renal domains. Beyond short-term weight reduction, these therapies have demonstrated durable effects on long-term metabolic health, cardiovascular resilience, and renal preservation, reinforcing their role in systemic disease modification rather than transient symptom management. In combination with other treatment modalities, such as lifestyle interventions, additional pharmacotherapies, and/or bariatric surgery, microdosing may better support patients in achieving their individual long-term health goals by providing multi-system health benefits and thus improving their quality of life. Of note, there is a limited direct evidence base for GLP-1 RA microdosing, and this off-label approach requires more direct studies to confirm that fractional doses maintain true long-term benefits across different body systems. As such, utilization of microdosing any medication should be approached with caution, and thorough discussion with a licensed provider should be done prior to initiating a microdosing regimen.

## References

[1] Celletti F, Farrar J, De Regil L (2026). World Health Organization guideline on the use and indications of glucagon-like peptide-1 therapies for the treatment of obesity in adults. JAMA.

[2] Fryar CD, Afful J, Saif NT (2026). Health E-Stat 111: Prevalence of Overweight, Obesity, and Severe Obesity Among Adults Age 20 and Older: United States, 1960-1962 Through August 2021-August 2023.

[3] Purnell J (2000). Definitions, classification, and epidemiology of obesity. Endotext [Internet].

[4] Noiman A, Fryar CD, Saif NT, Afful J (2026). Prevalence of Overweight, Obesity, and Severe Obesity Among Children and Adolescents Ages 2-19 Years: United States, 1963-1965 Through August 2021-August 2023.

[5] Nauck MA, Quast DR, Wefers J, Meier JJ (2021). GLP-1 receptor agonists in the treatment of type 2 diabetes - state-of-the-art. Mol Metab.

[6] Emmerich SD, Fryar CD, Stierman B, Ogden CL (2024). Obesity and severe obesity prevalence in adults: United States, August 2021-August 2023. NCHS Data Brief.

[7] Gudzune KA, Kushner RF (2024). Medications for obesity: a review. JAMA.

[8] Elmaleh-Sachs A, Schwartz JL, Bramante CT, Nicklas JM, Gudzune KA, Jay M (2023). Obesity management in adults: a review. JAMA.

[9] Lappin G, Noveck R, Burt T (2013). Microdosing and drug development: past, present and future. Expert Opin Drug Metab Toxicol.

[10] Wing RR (2021). Does lifestyle intervention improve health of adults with overweight/obesity and type 2 diabetes? Findings from the Look AHEAD Randomized Trial. Obesity (Silver Spring).

[11] Komé AM, Chandran MM, Tungate Lopez SS, Buse JB, Klein KR (2025). One size does not fit all: understanding microdosing semaglutide for diabetes in multidose pens. Diabetes Care.

[12] Neri LC, Mariotti F, Guglielmetti M, Fiorini S, Tagliabue A, Ferraris C (2024). Dropout in cognitive behavioral treatment in adults living with overweight and obesity: a systematic review. Front Nutr.

[13] Yeo D, Jo Y, Jeong J (2026). Efficacy and safety of glucagon-like peptide 1 receptor agonists across all health outcomes in type 2 diabetes: an umbrella review and evidence map of randomised controlled trials. Diabetes Obes Metab.

[14] Grunvald E, Shah R, Hernaez R (2022). AGA clinical practice guideline on pharmacological interventions for adults with obesity. Gastroenterology.

[15] Kong F, Zhao Y, Zhang W (2026). Comprehensive evaluation of GLP-1 receptor agonists: an umbrella review of clinical outcomes across multiple diseases. Nat Commun.

[16] Nathan PJ, O'Neill BV, Napolitano A, Bullmore ET (2011). Neuropsychiatric adverse effects of centrally acting antiobesity drugs. CNS Neurosci Ther.

[17] Flint A, Raben A, Astrup A, Holst JJ (1998). Glucagon-like peptide 1 promotes satiety and suppresses energy intake in humans. J Clin Invest.

[18] Kramer CK, Leitão CB, Pinto LC, Canani LH, Azevedo MJ, Gross JL (2011). Efficacy and safety of topiramate on weight loss: a meta-analysis of randomized controlled trials. Obes Rev.

[19] Galli M, Benenati S, Laudani C (2025). Cardiovascular effects and tolerability of GLP-1 receptor agonists: a systematic review and meta-analysis of 99,599 patients. J Am Coll Cardiol.

[20] Lee MM, Sattar N, Pop-Busui R (2025). Cardiovascular and kidney outcomes and mortality with long-acting injectable and oral glucagon-like peptide 1 receptor agonists in individuals with type 2 diabetes: a systematic review and meta-analysis of randomized trials. Diabetes Care.

[21] Newsome PN, Buchholtz K, Cusi K (2021). A placebo-controlled trial of subcutaneous semaglutide in nonalcoholic steatohepatitis. N Engl J Med.

[22] Rakipovski G, Rolin B, Nøhr J (2018). The GLP-1 analogs liraglutide and semaglutide reduce atherosclerosis in ApoE-/- and LDLr-/-) mice by a mechanism that includes inflammatory pathways. JACC Basic Transl Sci.

[23] Bicca J, Murta I (2025). SAT-685 low-dose tirzepatide for the treatment of patient with lipedema: a case report. J Endocr Soc.

[24] Reich-Schupke S, Schmeller W, Brauer WJ (2017). S1 guidelines: lipedema. J Dtsch Dermatol Ges.

[25] Masson W, Lobo M, Nogueira JP, Barbagelata L, Touzas P, Frías JP (2026). Anti-inflammatory effects of tirzepatide: a systematic review and meta-analysis. Rev Endocr Metab Disord.

[26] Cullinane PW, de Pablo Fernandez E, König A, Outeiro TF, Jaunmuktane Z, Warner TT (2023). Type 2 diabetes and Parkinson's disease: a focused review of current concepts. Mov Disord.

[27] Svenningsson P, Wirdefeldt K, Yin L, Fang F, Markaki I, Efendic S, Ludvigsson JF (2016). Reduced incidence of Parkinson's disease after dipeptidyl peptidase-4 inhibitors-a nationwide case-control study. Mov Disord.

[28] Michailidis M, Moraitou D, Tata DA, Kalinderi K, Papamitsou T, Papaliagkas V (2022). Alzheimer's disease as type 3 diabetes: common pathophysiological mechanisms between Alzheimer's disease and type 2 diabetes. Int J Mol Sci.

[29] Helal MM, AbouShawareb H, Abbas OH, Haddad R, Zain Y, Osman AS, Hassan AK (2025). GLP-1 receptor agonists in Parkinson's disease: an updated comprehensive systematic review with meta-analysis. Diabetol Metab Syndr.

[30] Hendricks RM, Khasawneh MT (2021). An investigation into the use and meaning of Parkinson's disease clinical scale scores. Parkinsons Dis.

[31] Yılmaz N, Bastemir M (2026). Gastrointestinal adverse effects of GLP-1 and dual GLP-1/GLP receptor agonists: a comprehensive update in diabetic and obese populations. Diabetes Metab Syndr Obes.

[32] Petri KC, Ingwersen SH, Flint A, Zacho J, Overgaard RV (2018). Exposure-response analysis for evaluation of semaglutide dose levels in type 2 diabetes. Diabetes Obes Metab.

[33] Peng X, Wang Z, Han K (2026). Super responders to biologic therapy in psoriasis: definitions, predictors, and implications for precision medicine. Front Immunol.

[34] Nadolsky K, Garvey WT, Agarwal M (2025). American Association of Clinical Endocrinology consensus statement: algorithm for the evaluation and treatment of adults with obesity/adiposity-based chronic disease - 2025 update. Endocr Pract.

[35] Nauck MA, Tuttle KR, Tschöp MH, Blüher M (2026). Glucagon-like receptor agonists and next-generation incretin-based medications: metabolic, cardiovascular, and renal benefits. Lancet.

[36] Melson E, Ashraf U, Papamargaritis D, Davies MJ (2025). What is the pipeline for future medications for obesity?. Int J Obes (Lond).

[37] Aronne L. J., Horn D. B., le Roux C. W. (2025). Tirzepatide as compared with semaglutide for the treatment of obesity. N Engl J Med.

[38] Hankosky ER, Chinthammit C, Meeks A, Huang A, Ward JM, Mojdami D, Gibble TH (2025). Real-world use and effectiveness of tirzepatide among individuals without type 2 diabetes: results from the Optum Market Clarity database. Diabetes Obes Metab.

[39] Angelopoulos N, Androulakis I, Rizoulis A (2026). A real-world study of tirzepatide for weight loss in adults without diabetes mellitus. Int J Obes (Lond).

